# Lectures on Some of the More Important Points in Surgery

**Published:** 1846-11

**Authors:** G. J. Guthrie


### RECORD OF MEDICAL SCIENCE.

*Lectures on some of the more Important Points in Surgery.* Delivered at the Royal Westminster Ophthalmic Hospital, Charing Cross. By G. J. Guthrie, F. R. S., &c.

LECTURE VIII.

*General Conclusions.*

1. The Hunterian operation for the cure of an aneurism is not applicable to the treatment of a wounded artery, inasmuch as the wound of the artery communicates with the external parts, and nothing intervenes to prevent blood flowing from the wound in its side, or from its cut extremities.

2. When a large artery is divided and bleeds, the wound should be enlarged if necessary, and a ligature placed on both the divided ends ; but if the artery be only injured and not quite divided, the ligatures should be applied one immediately above, the other below the injured part. The artery may or may not be then cut across, at the pleasure of the operator, but the limb or part should be placed in a relaxed position. A bandage should not be applied, and the edges of the wound should be simply brought together by adhesive plasters, which do not extend completely round the limb.

3. No operation is to be performed on any artery unless it bleeds at the moment of its performance, inasmuch as hemorrhage once suppressed may never return.

4. The intervention of muscular fibres, or of whole muscles, is not a sufficient reason for tying the artery at a distant part. They must be divided, if it be possible, to the extent required for a due exposure of the injured artery and its accompanying veins and nerves.

5. If the wound pass indirectly to the principal artery, from the back of the thigh for instance to the femoral artery in front, or from the outside of the arm to the humeral artery on the inside, the surgeon may (on satisfying himself of the part likely to be injured, by the introduction of a probe) cut down on a vessel opposite that supposed to be wounded, by the most simple and approved method. When the artery is exposed, the probe will point out the spot at which the vessel has in all probability been wounded. Pressure made below this spot on the artery, will cause it to be distended and to bleed, if the flow of blood be not prevented from above ; the artery is then to be secured by two ligatures, and the lower one should if possible be applied first.

6. The tourniquet should never be applied in an operation for aneurism or for a wounded artery. Compression by the hand in the course of the wounded vessel is allowable.

7. The blood from the upper end of a divided artery, or that nearer the heart, is of a scarlet arterial colour.

10. The blood from the lower end of a divided artery, or that which is further from the heart, is of a dark or venous colour, when it happens to flow immediately after the division of the vessel. At a subsequent period it may assume more of the colour of arterial blood, but it rarely does so for several days after the receipt of the injury, and always flows, or at least until a very late period, in a continued stream.

11. This regurgitation or flow of blood from the lower end of a divided artery is a favorable sign, inasmuch as it shows that the collateral circulation will probably be sufficient to maintain the life of the extremity.

12. The collateral circulation is in almost every instance capable of maintaining the life of the upper extremity when the axillary artery is divided, and the colour of the blood which flows from the end of the artery, on its being divided, is not always as dark as in the lower extremity, and it sooner resumes its arterial colour.

13. The collateral circulation is not always capable of maintaining the life of the limb when the femoral artery is injured. The best assistance which art can give is to rub the foot and leg in the gentlest manner, between the hands of one or two strong young women, for several hours, or even for the first three or four days; relaxing this process very little, even during sleep. When the vein is divided at the same time, or rendered impervious, the limb usually mortifies.

14. The collateral circulation is sufficient to maintain the life of an extremity in almost every case in which an aneurism has existed for eight or ten weeks, although it may be incapable of doing this if the principal artery has been suddenly divided, without any previous disease having existed in the part.

15. The theory and the operation for aneurism are never to be applied to the treatment of a wounded artery, which has caused a diffused or circumscribed aneurism, *whilst the external wound communicates with the artery*, unless it be impossible or impracticable to tie the bleeding vessel.

16. When an artery has been wounded, and the external opening has healed for weeks or months, so as to give rise to a diffused or circumscribed aneurism, it may be treated according to the theory of aneurism occuring from an internal cause, if the case will permit it without danger, although with this difference, that as the artery is sound the operation may be performed close to the tumour. If any doubt exist as to the capability of the collateral circulation to support the life of the lower extremity, when the external iliac is secured by ligature, the operation should be performed at the injured part by opening the swelling and enlarging the wound, as in the case of a wounded artery.

17. When a circumscribed or diffused aneurism has formed after a wound has been opened, whether by accident or design, it is placed in the situation of a wounded artery, and should be treated as such. If the aneurism has arisen from disease of the vessel, and the wound or opening into it cannot be permanently closed, the limb is in a worse state than if the artery had been wounded by accident; because a ligature or ligatures placed on a diseased artery are little likely to be successful. They are liable to all the difficulties and inconveniences attendant on the old operation for aneurism. If a case of the kind should occur in a popliteal or femoral aneurism, situated at or below where the artery passes between the triceps and the bone, amputation, if it can be done low down, will be the best remedy. If the aneurism should have formed higher up, and the opening can be closed with any prospect of its healing, a ligature may be placed upon the artery above it; but on the recurrence of hemorrhage which cannot be restrained by moderate pressure, the artery must be tied below, or recourse had to amputation. It is, however, to be observed, that amputation under these circumstances, when resorted to as a third operation, rarely succeeds.

18. When an artery is wounded with a simple fracture of a bone, or with a comminuted fracture of smaller bones, with an external communicating opening, both ends of the artery should be secured, and the limb treated in the usual manner.

19. When the bone broken is the femur, and the artery divided is the femoral artery, the operation of amputation will generally be advisable. It will always be so if the fracture is a comminuted one, or the shaft of the bone is extensively split.

20. When the broken bone injures the artery and gives rise to an aneurism, the treatment is to be first of the fracture and then of the aneurism, as soon as circumstances render it advisable or necessary to have recourse to the operation for aneurism, and which can only be after time has been given for the collateral branches to enlarge, so as to maintain the life of the limb.

21. When mortification takes place in addition to, or as a consequence of a wounded artery, amputation should be had recourse to forthwith.

22. The place of operation should be in almost all cases at the seat of the original wound; but there may be an exception, viz:

23. When the injury has been a mere cut, just sufficient to divide the artery and vein immediately below Poupart's ligament, and mortification of the foot supervenes, amputation should be performed below the knee, or at the part where the mortification more usually stops for a time.

This rule is founded on the observation, that great efforts are made by nature to arrest mortification a little below the knee. Sometimes they succeed; when they fail, death is almost inevitable. The advice to amputate at this part is founded on the fact of its being infinitely less dangerous, when done there, than on the thigh, independently of saving a joint.

24. When mortification has *continued for several days,* and is spreading without having once stopped, the constitution of the patient being implicated as marked by fever, amputation should not be performed until the mortification has been arrested and the line of separation has been well formed. In many cases, where there is great weakness or of irritability of constitution, it will be advisable to defer the operation to a later period, particularly if there be hope of the patient's becoming stronger and more tranquil.

25. If the mortification has once stopped and then begins again to spread, it will never again cease to extend, and amputation may give some chance of life.

26. Amputation of the arm should never be had recourse to, in consequence of a wound of the axillary artery, unless mortification takes place.

27. When mortification takes place after the operation for aneurism, the surgeon must be guided by the state of the patient's constitution, in resorting to or refraining from amputation.

28. When hemorrhage takes place from the surface of a stump, the artery should be tied at the part from which the blood comes in the first instance, if it can be easily done. If this should not suffice, the artery must be tied higher up, just at such distance as will afford a fair hope of its not having been affected by the derangement of the stump, which has led to the failure of consolidation in the extremity of the artery, and yet not too high to admit of the junction of any large collateral branches. If the bleeding proceeds from several small vessels, and cannot be arrested, the principal trunk should be tied above the diseased part, and the patient removed to a pure atmosphere, without which the operation rarely succeeds in any case.

29. When an aneurismal tumor mortifies, it is unnecessary and improper to tie the artery above the tumor, because it will be obliterated if the mortification be arrested by the efforts of nature, which the operation may interfere with, and even prevent, whilst, if the mortification spreads, it will be a matter of supererogation, and only hasten the patient's dissolution. When an aneurism inflames, is opened by ulceration, and bleeds profusely, it is a proper case for amputation, if such an operation can be performed.

*On the Operations of placing a Ligature on the Aorta, on the Common Trunk of the Iliac, and on the Internal and External Iliac Arteries.*

In performing either of the three operations, it is advisable to compute the point at which the artery is to be tied, with relation to the umbilicus and the anterior superior spinous process and the crest of the ilium. The aorta bifurcates usually on the body of the fourth, or on the intervertebral substance, between it and the fifth vertebra, although it may be higher or lower, which cannot be ascertained previously to the operation; the most usual place being nearly opposite to the margin of the umbilicus on the left side. It is about half an inch above this that the ligature should be placed on the aorta, if this operation is ever done again, rather lower than higher, on account of the origin of the inferior mesenteric artery. As this artery is to be reached by carrying the finger along the common iliac, the comparative situation of that vessel is next to be estimated.

The aorta divides into the two common iliac arteries, the length of which varies according to the stature of the patient, and the place at which the aorta bifurcates. The common iliacs again divide into the external and internal iliacs, which division is usually opposite to the sacro-iliac symphysis. The length of the common iliac artery is therefore tolerably well defined, as scarcely ever exceeding two inches and three quarters, and seldom being shorter than two inches. The external iliac is a little longer than the common iliac, and the place of subdivision of the common iliac into external and internal can always be ascertained during an operation, by tracing the external iliac upwards to its junction with the internal iliac to form the common trunk, which proceeds upwards and inwards to the aorta. The left margin of the umbilicus being taken as a point opposite to that which may be presumed to be the part at which the aorta divides, and the situation of the external iliac becoming femoral being clearly ascertained, a line drawn between the two will nearly indicate the course of these vessels: sufficiently so, at all events, to enable the operator to mark with his eye the place where he expects to tie the artery, and to regulate the length of the incision, so that this ideal spot may correspond to its centre. It is necessary to recollect also, that the whole of one hand and part of the other must be introduced into the wound, to enable tho operator to pass the ligature round the artery, and to tie the knots ; so that an external incision of less extent than five inches will not suffice, and six will afford a facility in operating, which will save pain to the patient, and inconvenience to the operator. In calculating the length of the incision, allowance must be made for the size, obesity, and muscularity of the patient. If a rule be placed on the crest of each ilium, about one inch and a half behind the anterior superior spinous process, it will pass in a well-formed man across the junction of the fifth lumbar vertebra with the upper part of the sacrum, and a little way behind where the common iliac divides into external and internal. The centre of an incision, six inches in length, beginning about half an inch above Poupart's ligament, and about the same distance to the outside of the inner ring, and carried upwards, will fall nearly on a line with this point. The incision should be nearly parallel to the course of the epigastric artery, but a little more to the outside, in order to avoid it and the spermatic chord, and having a gradual inclination inwards towards the external edge of the rectus muscle, the patient being on his back, with the head and shoulders raised, and the legs bent on the trunk. The aponeurosis of the external oblique muscle having been opened inferiorly, is to be slit up for the whole length of the external incision ; and the director having been first passed under the internal oblique muscle, through a small opening carefully made into it, it is to be divided in a similar manner. The transversalis is then to be cut through at the under part, and its tendinous expansion divided at the upper part, the greatest precaution being taken by the finger to prevent the peritoneum from being injured. The fascia transversalis is then to be torn through at the lower and outer part, so that the fingers may be passed outwards towards the ilium, and the perit neum detached from the iliac fossa, and turned with its contents inwards, by a gradual and sidelong movement of the fore and second finger inwards and upwards, until passing over the psoas muscle the external iliac artery is discovered by its pulsation. It is then to be traced upwards and inwards towards the spine, when the origin of it and the internal iliac from the common trunk will be felt. The point of the forefinger will then be nearly in the centre of a line drawn from the umbilicus to the anterior superior spine of the ilium ; and hence the necessity for an incision of six inches in length, if the artery is to be tied high up, which is to be accomplished by tracing it in a similar manner to its origin from the aorta.

If the *internal iliac* is to be tied, the operator traces it downwards from its origin, in preference to passing his finger from the external iliac artery inwards in search of it. Having placed the point of his fore finger on the vessel at the part where he intends to pass his ligature, he scratches with the nail upon and on each side of it, so as to separate it from its cellular attachments, and from the vein which accompanies but lies behind it. Thus far the operator proceeds by feeling; but it is now necessary that the sides of the wound should be separated, and kept apart by blunt spatulæ curved at the ends, so as to take up as little space as possible, and not injure the peritoneum. The surgeon should, if possible, see the artery, and the ligature carried on the eye of a bent probe, or a convenient aneurismal needle should be passed under it from within outwards, when it should be taken hold of with the forceps ; the probe or needle should then be withdrawn, and the ligature firmly tied twice, or with a double knot. Great care must be taken to avoid every thing but the artery. The peritoneum which covers it, and the ureter which crosses it, must be particularly kept in mind. The situation of the external iliac artery and vein, which have been crossed to reach it, must be always recollected, and if there be sufficient space they should be kept out of the way, and guarded by the finger of an assistant.

The *common trunk* of the iliac arteries and the aorta itself may be tied by the same method of proceeding; the only difference which can be practised with advantage will be to make the incision a little longer at its upper part; no inconvenience arising from the addition to the length of the external wound, whilst the subsequent steps of the operation are much facilitated by it. The following method of proceeding, adopted in cases 31 and 108, will bring the method of operating so graphically before the reader that it cannot be misunderstood, and may be readily followed in operating. I began the operation, the patient lying on the back, by an incision on the fore part of the abdomen, commencing an inch and a half below the inside of the anterior spine of the ilium, and the same distance within it, carrying it upwards, and diagonally inwards towards the edge of the rectus muscle above the umbilicus, so that the incision was between six and seven inches long. If the incision is made more outwardly, towards the side in a straight or vertical line from the ilium towards the ribs, great difficulty will be experienced in turning over the peritoneum with its contents, so as to place the finger on the last lumbar vertebra, an inconvenience which will be avoided by making the incision diagonally and of the length directed.

After dividing the common integuments, the three layers of muscles were cut through in the most careful manner; the division of the transversalis muscle was attended with some difficulty, inasmuch as there was little fascia transversalis, and the peritoneum was remarkably thin—as thin as white silver paper. On attempting to reach the under part on the inside of the ilium, so as to turn the peritoneum over, which in sound parts is always done without the least difficulty, I found that it could not be done on account of the tumor which projected inwards adhering to it, and some bleeding took place from the large veins which surrounded it, giving rise to the caution not to proceed further in that direction. At this moment, in spite of the greatest possible care that could be taken by Mr. Keate, who raised and protected the peritoneum, a very small nick was made in it, sufficient to show the intestine through it. Perceiving that I could not tie the internal iliac as I had at first intended, and that I must place the ligature on the common iliac, I tried to gain a greater extent of space upwards; but where the tendon of the transversalis muscle passes directly across from the lower ribs to and forming the sheath of the rectus, the peritoneum is usually so thin and so closely attached to it that it can be separated with great difficulty. I knew this from the operation I performed in case No. 50, when, in spite of all the precaution I could then take, the peritoneum was at this spot slightly opened. It occurred in the present instance, and the right lobe of the liver was thus exposed.

The opening thus made on the fore part of the abdomen was not large enough to admit two hands. The peritoneum being however separated a little from the posterior wall of the abdomen from the outside, four fingers of one hand were introduced beneath it, and it was turned a little over towards the opposite side. In doing this it must be remembered that the peritoneum must be raised, the hand being pushed towards the back as little as possible, in order to avoid getting behind the fat commonly found in that part of the body, which would lead to the under edge of the psoas muscle instead of the upper surface, and thus render the operation embarrassing.

The peritoneum being carefully drawn over with its contents, I found I could only get one hand, or a little more, underneath it in search of the artery, the tumors below preventing any further detachment of the peritoneum in that direction. I therefore passed my finger across the psoas muscle, and it rested on the fifth lumbar vertebra. The common iliac artery was not, however, to be felt even as high up as the fourth lumbar vertebræ, nor the aorta; they had both risen with the peritoneum, and my finger resting on the spine was beneath them. Mr. Keate endeavoured to raise or draw over the peritoneum, to give me an opportunity of seeing the vessels, but this was out of the question. In doing this, he felt the pulsation of the iliac artery, which had been raised with the peritoneum, to which I

found it adhering. Carefully separating it with the end of the fore finger of the right hand, I passed a single thread of strong dentists' silk, as it is termed, in a common solid aneurismal needle, by the aid of the thumb and fore finger of the left hand, round the artery without seeing it. I could bring the artery a little forward by means of the aneurismal needle, when it appeared to be perfectly clear, and from the distance of the bifurcation of the aorta above, I calculated that the common iliac was tied exactly at its middle part. All pulsation below immediately ceased.

The two ends of the ligature were twisted, the peritoneum replaced in its proper situation, care being taken that the two small openings into it should be well covered under the skin, so that they might not be in the line of the incision, and that they should be covered by partly divided healthy parts, which might thus adhere to each other. Three strong sutures and three or four smaller ones were put in first through the skin, in order to prevent the parts bursting asunder from the movements of the patient. This operation was only formidable from the circumstance, that space could not be obtained for the introduction of both hands, for, strange as it may appear, the safety of, and ease in doing, the operation depends on the first incision in the fore part of the abdomen being so large that the peritoneum containing the bowels may be freely drawn over by the expanded hands of the assistant, so that the operator can see what he is doing beneath. In case No. 107, the whole of the parts under the peritoneum could be seen distinctly, and several gentlemen not in the profession who were present, saw the common iliac artery in its natural situation. The aorta may be as readily tied by this mode of proceeding as the common iliac, and I am satisfied it is in this way such an operation ought to be performed, provided it becomes necessary to attempt it, which I suspect it will not be, for when an aneurism has been formed so high up that it prevents the application of a ligature on the side on which the disease is situated, the common iliac will be more readily tied above it, instead of the aorta, by performing the operation on the opposite or sound side of the body, for as a ligature can be applied with great ease on the sound side on the middle of the common iliac artery, it requires very little more knowledge and dexterity to pass over to the opposite or diseased side, and tie the artery above the aneurismal tumor, the size of which would have prevented the operation being done on its inner or affected side. The placing a ligature on the aorta for an aneurism in the pelvis will thus be rendered unnecessary; this is the most important result to be deduced from the operation described.

The patient suffered little or nothing from the operation, which was performed on Saturday; there was no augmentation of the pulse until Sunday evening, when it rose to 120; she then experienced some pain, which was materially diminished, although not altogether removed, by the abstraction of fourteen ounces of blood. At four o'clock in the morning, Mr. Hancock, now surgeon to the Charing-cross Hospital, took away fourteen ounces more, after which she had not a bad symptom. The bowels were not moved for the first four days. The temperature of the limb diminished, but not much, which may be attributed to a method adopted in case No. 108. For the first time two persons constantly rubbed the limb night and day, and a hot brick, in baths of hot water, covered by flannel, were applied to the feet, of the temperature of from 120° to 140°. One nurse rubbed the lower part of the limb, another the upper for three days and nights; if an interval of a few minutes elapsed a hot flannel was put on the limb. The friction was very slight so as not to injure the cuticle. The patient occasionally dozed a little, but the same gentle friction was kept up. The ligature came away on the twenty-sixth day after the operation. The external incision healed very readily, but was followed by a herniary projection, requiring the support of an abdominal bandage.

The situation of the ureter and rectum on the left side of this operation, and of the ureter and the cœcum with its appendix on the right side, should be well understood, and it should be known that the ureter rises with the peritoneum. The relative situation of the common iliac artery and vein should be particularly attended to in passing the ligature around the vessel. On the left side the artery lies external and anterior to its commencement: on the right, the artery passes over the commencement of the vena cava and the left iliac vein, which do not follow the peritoneum when drawn towards the opposite side. The bowels should be thoroughly well evacuated before the operation is performed, but purgatives should not be given for some days after it has been done. The food should be liquid, and inflammation should be subdued by leeches, general bleeding, fomentations, and opium.

The *external iliac* artery has been so often and so successfully tied that a description of the two methods of proceeding commonly adopted will suffice. The first, recommended by Mr. Abernethy, is in accordance with the operations on the common and on the internal iliac. The patient being laid on his back, with the shoulders slightly raised, and the legs bent on the trunk, an incision is to be made about three inches and a half in length in the direction of the artery, and terminating over or a little above Poupart's ligament. The aponeurosis of the external oblique muscle will be exposed, and an opening being made into it, a director is to be introduced, and it is to be slit up to the extent of the external incision. The internal oblique and transversalis muscles, are then to be "nicked," so as to allow a director or the point of the finger to be introduced below them, when they also are to be divided, the finger separating them from the fascia transversalis and peritoneum. The fascia transversalis running from Poupart's ligament to the peritoneum is now to be torn through with the nail, immediately over the pulsating artery, and the peritoneum is to be separated by the finger and pushed upwards until sufficient room is obtained; which, in this as well as in all other operations on the iliac arteries, is sometimes difficult on account of the protrusion of the intestines covered by the peritoneum, when the patient is not sufficiently tranquil. The artery is yet at some depth, and covered by a dense cellular membrane, connecting it to the vein on its inside, and which must be torn through with the nail. The anterior crural nerve is separated from the artery by the psoas muscle, at the outer edge of which it lies. The aneurismal needle should be passed between the vein and the artery, and the point made to appear on the outside of the artery.

In this operation the ligature is placed on the external iliac, above where it gives off the epigastric and circumflexa ilii arteries ; and as the operation is very much the same as that already described, with the exception of the incision being shorter and nearer to Poupart's ligament, it is obvious if it were found necessary from disease to tie the artery higher up, or even to tie the common iliac, that it might be done by merely enlarging the wound.

Another method has been recommended by Sir Astley Cooper, which is perhaps more followed where there is little doubt of the artery being sound. It offers the advantage of greater space, which enables the surgeon to see better what he is doing ; but it does not so readily admit of the artery being tied up, without the incision being extended upwards, so as to give more room for the introduction of the hand behind the peritoneum.

"The patient being placed in the recumbent posture, on a table of convenient height, the incision is to be begun within an inch of the anterior superior spinous process of the ilium, and it is to be extended downwards in a semicircular direction to the upper edge of Poupart's ligament. This incision exposes the tendon of the external oblique muscle : in the same direction the above tendon is to be cut through, and the lower edges of the internal oblique and transversalis abdominis muscles are exposed ; and the centre of these muscles is then to be raised from Poupart's ligament ; the opening by which the spermatic cord quits the abdomen is thus exposed, and the finger passed through this space is directly applied upon the iliac artery, above the origin of the epigastric and circumflexa ilii arteries. The iliac artery is placed upon the outer side of the vein ; and the next step in the operation consists in gently separating the vein from the artery by the extremity of a director, or by the end of the finger. The solid curved aneurismal needle is then passed under the artery, and between it and the vein from without inwards, carrying a ligature, which being brought out at the wound, the needle is withdrawn, and the ligature is then tied around the artery, as in the operation for popliteal aneurism. One end of the ligature being cut away, the other is suspended from the wound, the edges of which are brought together by adhesive plaster, and the wound is treated as any other containing a ligature."

This method of operating will suffice when the artery is to be tied for an aneurism which does not extend as high as Poupart's ligament. When it does, the operator will be so much inconvenienced by it, whilst the sound part of the artery above the tumour will be so much in a hollow behind it in the pelvis, that a ligature will not readily be passed around it, and the disturbance to the peritoneum will be much greater, and much more likely to give rise to peritonitis, than if the incision were made an inch longer on the face of the abdomen. The surgeon, instead of searching for the artery, as Sir Astley Cooper has directed, through the passage by which the spermatic cord quits the abdomen, and thus passing the fingers directly under the peritoneum, will find it very much for his own ease, and for the advantage of his patient, to pass his fingers under the peritoneum from the inside of the wall of the ilium, from which it readily separates, and thus approach the artery from the outside, instead of from below. He will gain more room, reach the artery easily above the origin of the circumflexa ilii, and avoid that disturbance of the peritoneum, in applying the ligature, which often leads to inflammation.

If the surgeon has unluckily divided the epigastric artery, either in this or any other operation, all that he has to do is to enlarge the incision, and tie both the divided ends; and I have no hesitation in saying, it will not be of any consequence, either in this operation or in one for hernia.

### *Of the Operation of placing a Ligature on the Gluteal and Sciatic Arteries.*

In all cases of aneurism of the gluteal and sciatic arteries, the internal iliac artery should be tied, instead of an operation on the part itself. In all cases of wounds of arteries which are the only ones rendering an operation for placing a ligature on these vessels necessary, the wound should in a great measure regulate the course of the incision. The operation is an act of simple dissection, first, through the common integuments for the space of four inches, then through and between the fibres of the glutæus muscle to the same extent; a dense aponeurosis covering the vessels is to be next divided, when the bleeding will lead to the injured vessel. In the dissecting-room the operation is described as follows:—Place the body on the face, turn the toes inwards; commence the incision one inch below the posterior spinous process, and one inch from the sacrum, carry it on towards the great trochanter in an oblique direction to the extent of four inches, divide the gluteus muscle and the aponeurosis beneath it, and seek for the artery as it escapes through the upper and anterior part of the sciatic notch, and lying close to the bone. If the vessels of the gluteus muscle bleed, so as to be troublesome, and cannot be stopped by compression, they must be secured.

If the sciatic artery be the vessel injured, the incision should be made in the same direction, but about an inch and a half lower down; if the course of the wound renders it doubtful which artery is wounded, the incision should be as nearly as possible between the two lines directed, the wound being always the best guide; and care should be taken in every instance to include nothing in the ligature but the artery.

*On the Operations on the Femoral Artery.*

Compression should never be made on an artery on which a ligature is about to be placed; because the pulsation is thereby suppressed, and the most important guide to the vessel is at the same time taken away. Where the artery is wounded and bleeding, compression must be had recourse to in the first instance to arrest it; and the first incision must be made without the information which the pulsation gives as to the precise situation of the artery, although the finger may be allowed to rest on the part, underneath which the artery could be felt before the pressure was applied. The external incision should always be made longer or shorter in proportion to the depth at which the artery is situated. It should be at least one-third longer in the middle than at the upper part of the thigh; and whilst a long incision always facilitates the subsequent steps of the operation, it never does harm, unless it be out of all reasonable proportion. The centre of the incision should be if possible directly over that part of the artery on which it is intended to apply the ligature; but no inconvenience will arise from its being applied nearer its upper extremity. The patient being laid on his back, and properly supported, the knee is to be bent and turned outwards, by which the head of the femur will be rolled in the acetabulum, and the femoral artery will be more distinctly felt at the upper part lying on the psoas muscle, having the vein to the inside of it, and the anterior crural nerve about half an inch on its outside, passing between the psoas and iliacus muscles, although some branches soon approach the artery, and run down on the external part of the sheath. The relative position of the parts being duly considered, an incision is to be made directly in a line over the pulsating artery, and carried through the skin, cellular tissue, and superficial fascia, down to the deep-seated, or fascia lata of the thigh. If an absorbent gland should be in the way, it must be turned aside or removed. The arteria profunda femoris is given off about two inches below Poupart's ligament, on the back part and outside, whilst three or four small vessels spring from half an inch to an inch below it on the forepart, and one or other of these may be divided. They are the superficial epigastric, the superficial pudic, the superficial circumflex of the ilium, and probably an artery supplying the absorbent glands. If they bleed so as to be troublesome they must be secured, more particularly if the femoral artery is to be tied below them. The fascia lata is now to be divided, with that part of the fascia transversalis, which descending beneath Poupart's ligament forms the sheath of the artery, when the vessel will be exposed. In dividing this fascia and sheath, the point of the knife is always to be directed to the centre of the artery, so that if it be cut by accident it may be seen, when the only result will be the necessity for the application of a ligature above, and one below it. The artery being fully exposed, as ascertained by the pulsation being felt by the finger, it is to be separated from its cellular attachment to the sheath on each side by a blunt or silver knife; and the aneurismal needle or probe, armed with a strong single thread of dentists' silk, is to be passed under it from the inner or pubic side outwards, by which all injury to the vein from the round point of the needle or probe will be avoided. Two common knots are to be made in the usual manner, when one thread may be cut off, or the two twisted together and brought carefully out of the wound; the edges of which are then to be duly approximated and retained in that situation by sticking plaster and a moderate compress, also secured in a similar manner. The knee is to be bent forward to relax the parts, and laid on the outside with a pillow underneath it.

The needle will pass more easily under the artery if the thigh is bent on the trunk ; and before the knots are tied, the surgeon should ascertain that pressure on the part or artery which he has nearly surrounded by the ligature, suppresses the pulsation in the tumour below.

The operation for popliteal aneurism lower down in the thigh is to be done in the following manner :—

The surgeon having turned the knee outwards, and bent the leg inwards into the tailor's sitting position, to show the course of the sartorius muscle, should trace the artery from the groin downwards, until it appears to pass under that muscle. The external incision, four inches in length, made in the course of the artery, should pass over this point one inch, so that when the fascia lata is divided, the sartorius muscle may be seen crossing over to the inside at the lower extremity of the wound. The fascia lata is to be divided for the space of two inches of the incision upwards. The fore-finger is then to be introduced into the wound, and pressure made with it rather outwardly, when it will readily distinguish the pulsation of the artery, still included in its sheath. This is to be opened by slight and repeated touches of the knife directly over the centre of the line of the vessel, or it may be divided on the director, when the artery will be exposed. The point of the fore-finger will easily recognise it from the roundness and firmness of the feeling communicated by it as well as by its pulsation, and the end of the nail, or handle of the scalpel or blunt knife, will separate it with facility from its attachments, to such an extent as will admit the blunt point of the solid unyielding aneurism needle to be passed beneath it from the pubic side. If the point of the needle does not readily come through the cellular attachments of the artery on the outside, this part must be touched lightly with the scalpel, or rubbed with the nail until the ligature is exposed, which should then be taken hold of with the forceps, and one end drawn out, whilst the instrument with the other end is withdrawn. The operator, taking both ends of the ligature, which has been in this manner passed under the artery, between the fingers of one hand, presses upon the artery, with the fore-finger of the other, so as to arrest the course of the blood in it, when if there be an aneurism below, the pulsation in it will cease. The ligature is then to be pressed upwards as far as the artery has been detached, and is to be tied with a double knot. The wound is to be dressed as in the previous case by adhesive plaster and compress, but without a bandage; and the patient is to be placed in bed, with his knee bent forward, or resting on the outside if more agreeable to him,

The operation is done in this manner on that part of the femoral artery which is not covered by muscle, and all interference with the sartorius is avoided. It is the improvement on the Hunterian operation recommended by Scarpa, and ought always to be adopted. This method obviates all discussion as to placing the ligature on the outside of the sartorius muscle; or as to the fear of injuring the absorbents; and as to the saphena vein, it can always be seen, and its course traced up the thigh and avoided. After the first incision is made and completed down to the fascia lata, that part is to be divided, I have said for the length of two inches, but this must be dependent on circumstances; the object being to obtain a view of the sheath containing the artery, the opening into which after the first touch of the knife may be completed with the assistance of the director underneath it; and the artery will be less disturbed in its lateral attachments by an opening into the sheath of three quarters of an inch in length, than by one of half the extent, as it will admit of the aneurism needle being passed under it with more facility, and consequently with less disturbance to the surrounding parts. I have never had reason to believe that a free opening into the fasica of the thigh has done mischief, or even into the sheath, provided the artery has not been unnecessarily disturbed.

The warmth of the limb operated upon should be maintained by gentle friction from the toes upwards to the knee, and when left at rest it should be enveloped in flannel. The wound should not be dressed until the fourth day, the limb being kept quite quiet; the patient should move as little as possible in bed, and the part of the heel on which it rests should be examined from time to time, as it may under pressure become gangrenous.

Suppression of the secretion of urine is not uncommon during the first twenty-four hours, and will be gradually removed by the patient's taking mild diluent drinks. The constitutional irritation in all these operations is frequently great, the pulse rising in forty-eight hours from eighty-five to one hundred and twenty; and if this continue until the third day, when the fear of mortification will have subsided, it should be moderated by the abstraction of a small quantity of blood. In cases of this kind I have had occasion to bleed twice, and with the happiest effect, the pulse having fallen in consequence to its natural standard. The medicines given at the same time were saline draughts every six hours, with four drops of Battley's solution of opium. The ligatures came away on and about the fifteenth day.

*London Medical Times.*

